# Accuracy of the END-PAC Model in Predicting the Risk of Developing Pancreatic Cancer in Patients with New-Onset Diabetes: A Systematic Review and Meta-Analysis

**DOI:** 10.3390/biomedicines11113040

**Published:** 2023-11-14

**Authors:** Shahab Hajibandeh, Christina Intrator, Eliot Carrington-Windo, Rhodri James, Ioan Hughes, Shahin Hajibandeh, Thomas Satyadas

**Affiliations:** 1 Department of General Surgery, University Hospital of Wales, Cardiff & Vale NHS Trust, Cardiff CF14 4XW, UK; eliot.carrington-windo@wales.nhs.uk (E.C.-W.); rhodri.james2@wales.nhs.uk (R.J.); hughes.ioan@yahoo.co.uk (I.H.); 2 Department of Hepatobiliary and Pancreatic Surgery, Manchester Royal Infirmary Hospital, Manchester M13 9WL, UK; intratorc@gmail.com (C.I.); tomsaty@yahoo.co.uk (T.S.); 3 Department of Hepatobiliary and Pancreatic Surgery, University Hospital Coventry & Warwickshire, Coventry CV2 2DX, UK; shahin_hajibandeh@yahoo.com

**Keywords:** pancreatic cancer, new onset diabetes, END-PAC

## Abstract

Objectives: To investigate the performance of the END-PAC model in predicting pancreatic cancer risk in individuals with new-onset diabetes (NOD). Methods: The PRISMA statement standards were followed to conduct a systematic review. All studies investigating the performance of the END-PAC model in predicting pancreatic cancer risk in individuals with NOD were included. Two-by-two tables, coupled forest plots and summary receiver operating characteristic plots were constructed using the number of true positives, false negatives, true negatives and false positives. Diagnostic random effects models were used to estimate summary sensitivity and specificity points. Results: A total of 26,752 individuals from four studies were included. The median follow-up was 3 years and the pooled risk of pancreatic cancer was 0.8% (95% CI 0.6–1.0%). END-PAC score ≥ 3, which classifies the patients as high risk, was associated with better predictive performance (sensitivity: 55.8% (43.9–67%); specificity: 82.0% (76.4–86.5%)) in comparison with END-PAC score 1–2 (sensitivity: 22.2% (16.6–29.2%); specificity: 69.9% (67.3–72.4%)) and END-PAC score < 1 (sensitivity: 18.0% (12.8–24.6%); specificity: 50.9% (48.6–53.2%)) which classify the patients as intermediate and low risk, respectively. The evidence quality was judged to be moderate to high. Conclusions: END-PAC is a promising model for predicting pancreatic cancer risk in individuals with NOD. The score ≥3 should be considered as optimum cut-off value. More studies are needed to assess whether it could improve early pancreatic cancer detection rate, pancreatic cancer re-section rate, and pancreatic cancer treatment outcomes.

## 1. Introduction

Pancreatic cancer is the fourth leading cause of cancer-related deaths in most developed countries including the European Union [1], with a 5-year survival of only 9% [2]. This uncommon cancer, whose annual incidence increases with age (37 in 100,000 in over-50-year-olds) [3], has poor survival rates due to its advanced stage at diagnosis. Earlier detection of resectable pancreatic cancer is crucial to improve survival [4]. However, due to its low incidence, government guidance, such as United States Preventive Services Taskforce, still advises against general population-based screening for pancreatic cancer [5]. Thus, identifying high-risk subgroups of patients who may benefit from targeted screening for early detection of sporadic pancreatic cancer has become of increasing value.

Several modifiable and non-modifiable risk factors are associated with pancreatic cancer such as smoking, alcohol, obesity and diet, as well as increasing age, gender, genetics and family history [2]. New-onset diabetes (NOD) after 50 years of age is a well-recognised, independent risk factor for developing sporadic pancreatic cancer, accounting for at least half of newly diagnosed cases [4]. Incidence of pancreatic cancer in patients with NOD over 50 years is approximately 1% within 3 years, a 6 to 8-fold increase from a risk in the general population of approximately 0.11% [3,4]. NOD is thought to be a paraneoplastic process that begins as early as 3 years before initial disease symptoms and differs from typical type 2 diabetes [6]. Further risk stratification amongst patients with NOD is required to identify those who would benefit most from screening as this involves resource-limited investigations [7].

Predictive models using different clinical features have been developed over the years. The Enriching New-Onset Diabetes for Pancreatic Cancer (END-PAC) model, developed by Sharma et al. [3], was able to achieve reasonable enough accuracy to generate interest in its use as a potential risk stratification tool [3,8]. The model uses only three parameters: age at diagnosis of NOD, change in weight and change in blood glucose from preceding year, differentiating these patients from those with type 2 diabetes [9]. The model classifies patients into low, intermediate and high-risk groups for developing pancreatic cancer; patients with an END-PAC score of three or more have a significantly higher risk of developing pancreatic cancer (3-year incidence of 3.6%) and warrant further investigations [3]. Exceptions to this include those who have active malignancy, severe illness, acute steroid use or rapid weight gain [3]. A main limitation of this model was that it was validated on an internal population with similar demographics and clinical care to the development cohort. Thus, external validation is crucial before incorporating END-PAC as a confirmed stratification tool into clinical practice [8].

Ultimately there is a strong need for a validated screening tool to predict which patients have a high risk of developing pancreatic cancer. Given the promising performance of the END-PAC model, we were encouraged to conduct a systematic review of the literature to assess its accuracy in predicting the risk of pancreatic cancer in patients with NOD.

## 2. Methods

The predefined protocol and design of this study were compliant with Preferred Reporting Items for Systematic reviews and Meta-Analyses (PRISMA) statement standards [10].

### 2.1. Eligibility Criteria

**Study design of interest**. All retrospective or prospective observational studies (cohort or case-control studies) and randomised controlled trials investigating the predictive value of the END-PAC model in determining pancreatic cancer risk in individuals with NOD were considered eligible. Case reports, correspondences, letters, review articles, systematic reviews and meta-analyses were excluded.

**Population of interest**. All patients aged above 50 with new-onset diabetes were considered eligible for inclusion.

**Predictive Model of interest**. The Enriching New-Onset Diabetes for Pancreatic Cancer (END-PAC) model as defined by Sharma et al. [3], comprising age at diagnosis of NOD, change in weight and change in blood glucose from preceding year, was the model of interest for inclusion.

**Outcome of interest**. The risk of pancreatic cancer during follow-up of the patients with NOD was considered as the outcome of interest. The predictive value of END-PAC in determining the risk of pancreatic cancer in individuals classed as high risk (END-PAC score ≥ 3), intermediate risk (END-PAC score 1–2), and low risk (END-PAC score < 1) by the model was evaluated.

### 2.2. Search Methods

Appendix A highlights the detail of the search strategy and electronic sources used. Two authors with expertise in evidence synthesis developed and incorporated the search strategy. The last search was applied on 30 June 2023 without any language restrictions.

### 2.3. Selection of Studies and Data Extraction

Two separate authors reviewed the title and abstract of the articles, obtained the full texts of potentially eligible articles and included the eligible studies. The following relevant data from the eligible studies were recorded on an electronic data collection sheet: bibliographic data, study design, sample size, included population description, follow-up, risk of pancreatic cancer, number of patients classed as high risk, intermediate risk, and low risk by the END-PAC model and number of true positives, true negatives, false positives and false negatives.

### 2.4. Risk of Bias Assessment

Two review authors independently used the Quality Assessment of Diagnostic Accuracy Studies 2 (QUADAS-2) criteria to assess the quality of the included studies [11]. The tool evaluates the risk of bias related to patient selection, index model, reference standards and flow and timing. The tool also evaluates the applicability concerns related to patient selection, index model and reference standard. We resolved disagreements between the first two review authors by discussion with a third review author.

### 2.5. Statistical Analyses

Two-by-two tables based on the reported number of true positives, false negatives, true negatives and false positives in included studies were constructed for patients classed as high risk (END-PAC score ≥ 3), intermediate risk (END-PAC score 1–2) and low risk (END-PAC score < 1) by the model. Subsequently, the coupled forest plots of the estimated sensitivities, specificities and their 95% confidence intervals (CIs) and the summary receiver operating characteristic (SROC) plots were constructed using Review Manager 5. We estimated the summary sensitivity and specificity point for each subgroup via a diagnostic random effects model using OpenMeta [Analyst] software (open-source version, Brown University, 2012). We evaluated the statistical heterogeneity by calculating I^2^ using the Cochran Q test (χ2) (low heterogeneity = I^2^ 0–25%; moderate heterogeneity = I^2^ 25–75%; high heterogeneity = I^2^ 75–100%).

### 2.6. Subgroup Analysis

We performed subgroup analyses based on the patients who were classed as high risk (END-PAC score ≥ 3), intermediate risk (END-PAC score 1–2), and low risk (END-PAC score < 1) by the END-PAC model.

### 2.7. Sensitivity Analyses

We performed sensitivity analyses based on the studies that were associated with low risk of bias and low applicability concerns based on the QUADAS-2 tool. Moreover, we planned to evaluate the effect of each study on overall results and heterogeneity by removing one study at a time.

## 3. Results

### 3.1. Results of the Search

**Figure 1:**
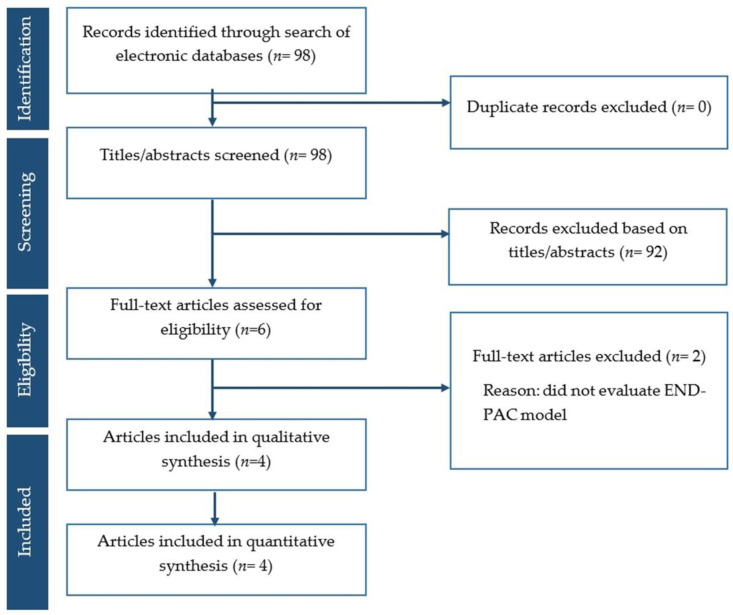
PRISMA flow chart.

The search of the literature yielded 98 articles; 92 studies were excluded due to irrelevance. After full text review, two more articles were excluded because they did not evaluate the END-PAC model. Consequently, four studies [3,8,9,12] comprising 26,752 patients were included (Figure 1). All of the included studies had retrospective design and included patients aged above 50 with NOD (Table 1). Among 26,752 patients with NOD, 215 patients developed pancreatic cancer, resulting in a pooled risk of 0.8% (95% CI 0.6–1.0) with a mean and median length of follow-up of 3.3 years and 3 years, respectively.

### 3.2. Risk of Bias Assessment

**Figure 2:**
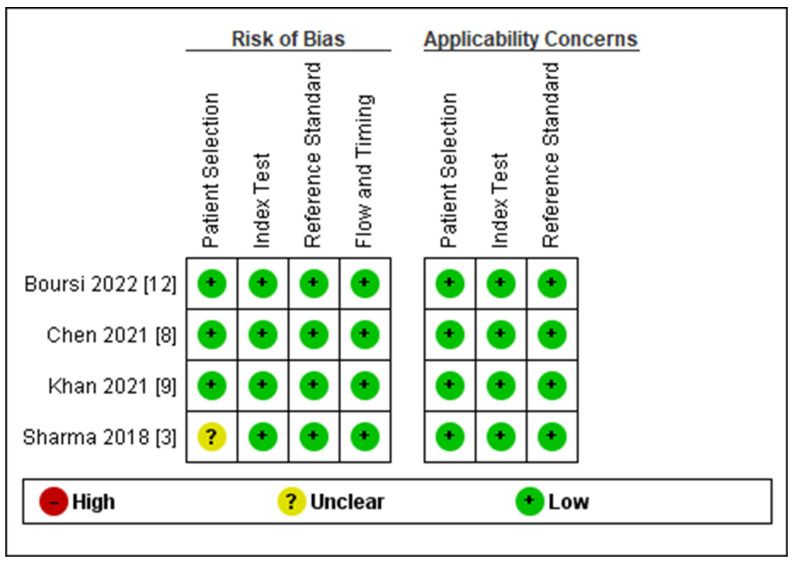

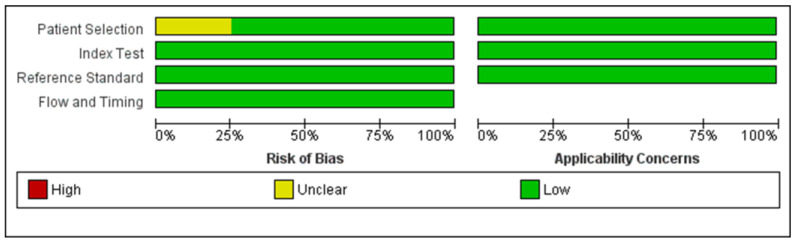
The risk of bias and applicability concerns summary and graph based on the QUADAS-2 criteria [3,8,9,12].

The risk of bias and applicability concerns summary and graph based on the QUADAS-2 criteria are demonstrated in Figure 2. The risk of bias related to patient selection was judged to be low in three studies and unclear in one study. The risks of bias related to index model, reference standard, and flow and timing were judged to be low in all of the included studies. There were no applicability concerns in terms of patient selection, index model, and reference standard in the included studies.

### 3.3. Performance of the END-PAC Model

**Figure 3:**
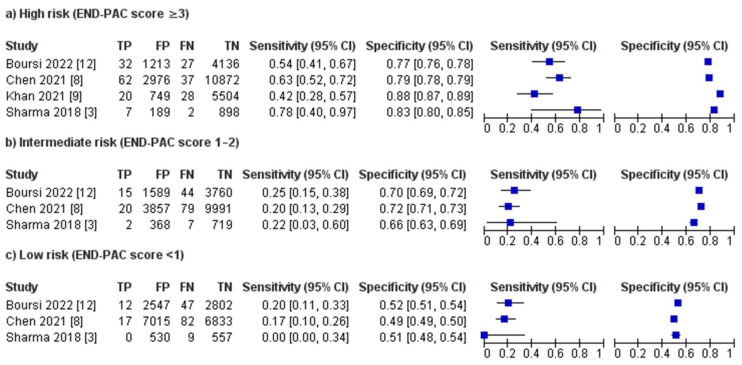
Coupled forest plots of the estimated sensitivities and specificities from individual studies for (**a**) high risk (END-PAC score ≥ 3), (**b**) intermediate risk (END-PAC score 1–2) and (**c**) low risk (END-PAC score < 1) [3,8,9,12].

**Figure 4:**
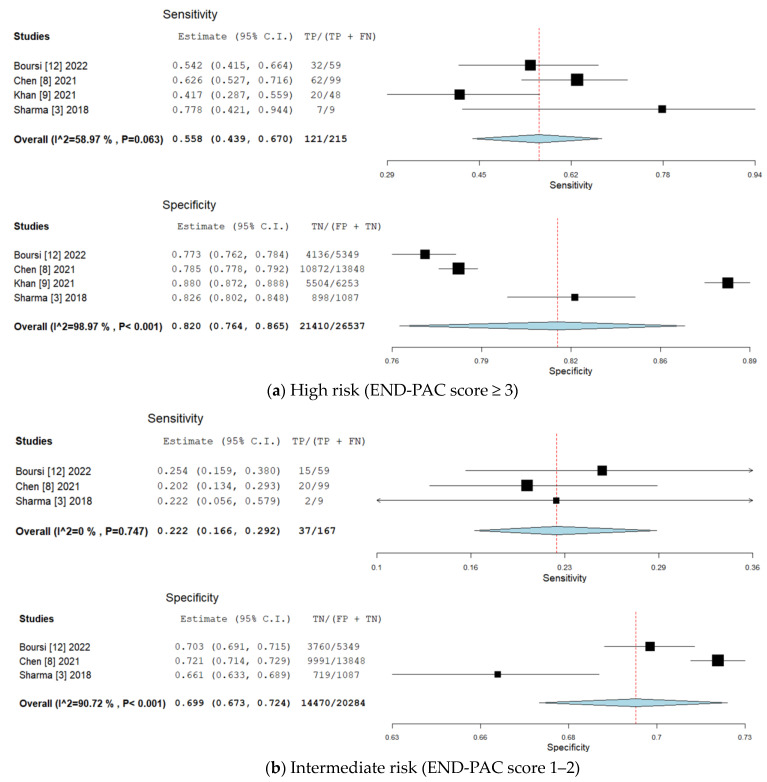

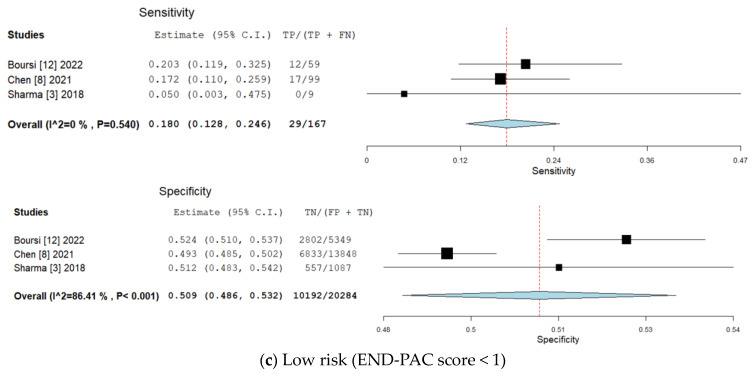
Forest plots for a summary sensitivity and specificity point for (**a**) high risk (END-PAC score ≥ 3), (**b**) intermediate risk (END-PAC score 1–2) and (**c**) low risk (END-PAC score < 1) [3,8,9,12].

**Figure 5:**
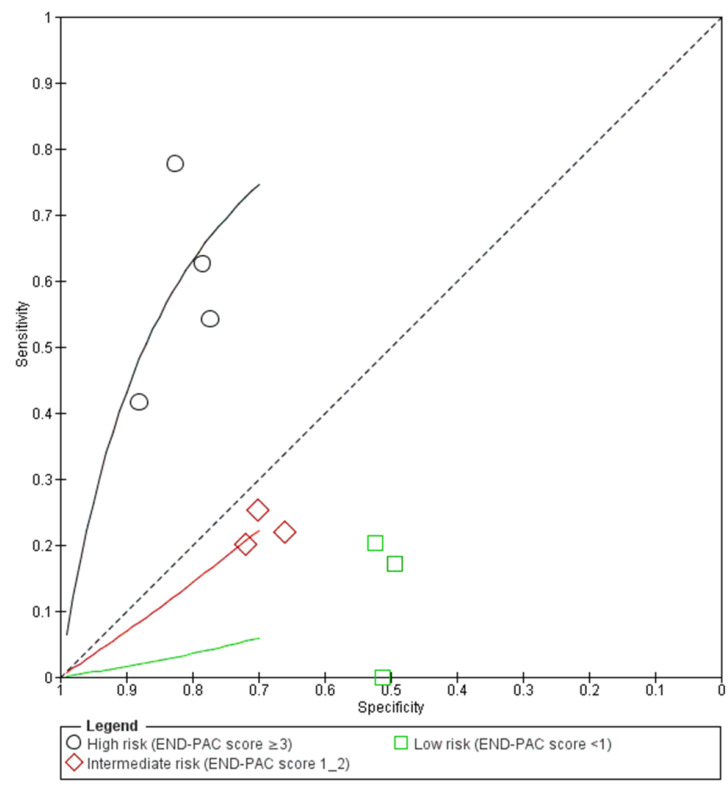
Summary ROC Plot of the following groups: high risk (END-PAC score ≥ 3), intermediate risk (END-PAC score 1–2) and low risk (END-PAC score < 1).

**High risk** (**END-PAC score ≥ 3**). Four studies including 26,752 patients evaluated the accuracy of the END-PAC model in patients classed as high-risk patients (END-PAC score ≥ 3) by the model. END-PAC score ≥ 3 was associated with 121 true positives, 5127 false positives, 21,410 true negatives and 94 false negatives. The visual assessment of the coupled forest plots of the estimated sensitivities and specificities suggested moderate between-study heterogeneity (Figure 3). END-PAC score ≥ 3 was associated with the pooled sensitivity of 55.8% (95% CI 43.9–67, I^2^ = 59%) and pooled specificity of 82.0% (95% CI 76.4–86.5, I^2^ = 99%) (Figure 4). Visual assessment of the SROC plot suggested good discrimination; END-PAC score ≥ 3 had significantly better discrimination than END-PAC score 1–2 or END-PAC score < 1 (Figure 5).

**Intermediate risk** (**END-PAC score 1–2**)**.** Three studies including 20,451 patients evaluated the accuracy of the END-PAC model in patients classed as intermediate-risk patients (END-PAC score 1–2) by the model. END-PAC score 1–2 was associated with 32 true positives, 5814 false positives, 14,470 true negatives and 130 false negatives. The visual assessment of the coupled forest plots of the estimated sensitivities and specificities suggested moderate between-study heterogeneity (Figure 3). END-PAC score 1–2 was associated with the pooled sensitivity of 22.2% (95% CI 16.6–29.2, I^2^ = 0%) and pooled specificity of 69.9% (95% CI 67.3–72.4, I^2^ = 91%) (Figure 4). Visual assessment of the SROC plot suggested suboptimal discrimination; END-PAC score 1–2 had lower discrimination than END-PAC score ≥ 3 but higher discrimination than END-PAC score < 1 (Figure 5). 

**Low risk** (**END-PAC score < 1**). Three studies including 20,451 patients evaluated the accuracy of the END-PAC model in patients classed as low-risk patients (END-PAC score < 1) by the model. END-PAC score < 1 was associated with 29 true positives, 10,092 false positives, 10,192 true negatives and 138 false negatives. The visual assessment of the coupled forest plots of the estimated sensitivities and specificities suggested moderate between-study heterogeneity (Figure 3). END-PAC score < 1 was associated with the pooled sensitivity of 18.0% (95% CI 12.8–24.6, I^2^ = 0%) and pooled specificity of 50.9% (95% CI 48.6–53.2, I^2^ = 86%) (Figure 4). Visual assessment of the SROC plot suggested suboptimal discrimination; END-PAC score < 1 had lower discrimination than END-PAC score ≥ 3 and END-PAC score 1–2 (Figure 5).

### 3.4. Sensitivity Analyses

The sensitivity analyses based on the studies with low risk of bias and low applicability concerns showed consistency of the results. Moreover, the overall results and heterogeneity remained unchanged after removing one study at a time.

## 4. Discussion

We conducted a systematic review and meta-analysis to investigate the performance of the END-PAC model in predicting pancreatic cancer risk in individuals with NOD. Analysis of 26,752 individuals from four studies suggested that END-PAC score ≥ 3 (high risk) is an optimum cut-off value for the model with promising sensitivity, specificity and accuracy in predicting the risk of pancreatic cancer; however, END-PAC score 1–2 (intermediate risk) and END-PAC score < 1 (low risk) were associated with suboptimal predictive performance. The clinical between-study heterogeneity was low and statistical between-study heterogeneity was moderate. The quality of the available evidence was judged to be moderate to high based on QUADAS-2 criteria.

Our study is the first systematic review and meta-analysis analysing the accuracy of the END-PAC model, which was first developed by Sharma et al. [3] and later reassessed in subsequent validation studies [8,9,12]. Considering that there is no previous systematic review on the accuracy of the END-PAC model to compare our findings with, we can compare the accuracy of the END-PAC model with other similar models. Boursi et al. [13] developed a predictive model in 2017 comprising a combination of clinical parameters (age, body mass index, change in body mass index, smoking, use of proton pump inhibitors and anti-diabetic medications) and laboratory parameters (haemoglobin, creatinine, glycosylated haemoglobin, alkaline phosphatase and cholesterol) [13]. The pooled sensitivity and specificity of END-PAC score ≥ 3 found in our study were comparable to those of the Boursi model (55.8% and 82.0% versus 44.7% and 94.0%, respectively). Both models were found to be encouraging in terms of narrowing down the screening population, but they were limited by their sensitivity [14]. The pooled sensitivity of END-PAC score ≥ 3 was 55.8%; however, the sensitivities reported by the included studies ranged between 42% and 78%. Therefore, the certainty of evidence about this finding is moderate and is downgraded due to heterogeneity in the reported sensitivities. Several prospective studies are underway, such as the NODES Trial, which is following up NOD patients over 60 years of age with clinical parameters and biomarkers [15]. The Early Detection Initiative is a prospective study run by Pancreatic Cancer Action Network to evaluate outcomes of a screening strategy using the END-PAC model and their results are highly anticipated [16].

The END-PAC model uses a patient’s age at diagnosis, change in body weight and change in fasting plasma glucose [3]. It is one of the first non-invasive computational methods to predict the risk of pancreatic cancer at an earlier stage. The model has a strong clinical rationale and is based on the paradoxical phenomenon linking pancreatic cancer-related NOD with weight loss versus weight gain in new onset type 2 diabetes mellitus [3]. For this reason, the model maintains sensitivity, even for patients developing pancreatic cancer-related NOD more than 12 months before clinical diagnosis. The second rationale relies on the difference in onset of glycaemic dysregulation in pancreatic cancer-related NOD, usually occurring over 2–3 years, much faster than in type 2 diabetes mellitus, in which they usually occur over 8 years [3]. The END-PAC model showed that fasting blood glucose and estimated average glucose were interchangeable in their use in the model and that the higher value of the two should be used [3].

The END-PAC model was designed to be part of a multi-step process for pancreatic cancer screening and highlights that patients with END-PAC score of 3 or above should undergo further imaging such as CT scan, MRI or endoscopic ultrasound. Schwartz et al. [17] found that the END-PAC model is straightforward to use, uses readily available clinical factors making patient workup easy, and is likely to be cost-effective, as CT imaging is a highly accessible screening modality [17]. The results of prospective studies such as the Early Detection Initiative would help to determine the true benefit of the END-PAC model.

The strengths of the current study include the following: (1) relatively large sample size in terms of the number of included patients; (2) similar baseline characteristics of the included studies; (3) low clinical between-study heterogeneity; and (4) moderate-to-high quality of evidence. We acknowledge there are limitations with the current study: (1) only four available studies on the topic of interest; (2) moderate statistical between-study heterogeneity; (3) publication bias could not be assessed because the number of included studies did not reach the minimum of 10; and (4) the retrospective nature of included studies, with the inevitable risk of selection bias.

## 5. Conclusions

END-PAC is a promising model for predicting pancreatic cancer risk in individuals with NOD. A score ≥ 3 should be considered as the optimum cut-off value. More studies are needed to assess whether it could improve early pancreatic cancer detection rate, pancreatic cancer resection rate and pancreatic cancer treatment outcomes.

## Figures and Tables

**Table 1:** Baseline characteristics of the included studies.

Study	Year	Country	Journal	Study Design	Length of Follow-Up	Description of Included Population	Model Evaluated	Model Variables	Sample Size
Boursi et al. [12]	2022	Israel	Pancreas	Retrospective cohort	3 years	Patients aged over 50 with new-onset diabetes	END-PAC	Age at diagnosis, change in body weight (kg), and change in fasting plasma glucose	5408
Khan et al. [9]	2021	USA	Pancreatology	Retrospective cohort	4 years	Patients aged over 50 with new-onset diabetes	END-PAC	Age at diagnosis, change in body weight (kg), and change in fasting plasma glucose	6301
Chen et al. [8]	2021	USA	Digestive Diseases and Sciences	Retrospective cohort	3 years	Patients aged between 50–85 with new-onset diabetes	END-PAC	Age at diagnosis, change in body weight (kg), and change in fasting plasma glucose	13,947
Sharma et al. [3]	2018	USA	Gastroenterology	Retrospective cohort	3 years	Patients aged over 50 with new-onset diabetes	END-PAC	Age at diagnosis, change in body weight (kg), and change in fasting plasma glucose	1096

END-PAC: Enriching New-Onset Diabetes for Pancreatic Cancer.

## Appendix A

**Table A1:** Literature search strategy and electronic resources used.

**Search No**	**Search Strategy ^†^**
#1	new-onset near 2 diabetes: TI, AB, KW
#2	MeSH descriptor: [pancreatic cancer] explode all trees
#3	pancreatic cancer: TI, AB, KW
#4	#2 OR #3
#5	ENDPAC: TI, AB, KW
#6	END-PAC: TI, AB, KW
#7	model: TI, AB, KW
#8	#5 OR #6 OR #7
#9	#1 AND #4 AND #8

Electronic databases used: Medical Literature Analysis and Retrieval System Online (MEDLINE), Cumulative Index to Nursing and Allied Health Literature (CINAHL), Excerpta Medica database (EMBASE), Cochrane Central Register of Controlled Trials (CENTRAL), Scopus, European Association for Grey Literature Exploitation, System for Information on Grey Literature, World Health Organization International Clinical Trials Registry, International Standard Randomised Controlled Trial Number Registry and ClinicalTrials.gov, and reference lists of relevant original studies, systematic reviews and meta-analyses.^†^ This search strategy was adopted for following databases: CINAHL, EMBASE, MEDLINE, CENTRAL and Scopus.

## References

[1] Barros A.G., Pulido C.F., Machado M., Brito M.J., Couto N., Sousa O., Melo S.A., Mansinho H. (2021). Treatment optimization of locally advanced and metastatic pancreatic cancer. Int. J. Oncol..

[2] Rahib L., Smith B.D., Aizenberg R., Rosenzweig A.B., Fleshman J.M., Matrisian L.M. (2014). Projecting cancer incidence and deaths to 2030: The unexpected burden of thyroid, liver, and pancreas cancers in the United States. Cancer Res..

[3] Sharma A., Kandlakunta H., Nagpal S.J., Feng Z., Hoos W., Petersen G.M., Chari S.T. (2018). Model to determine risk of pancreatic cancer in patients with new-onset diabetes. Gastroenterology.

[4] Chari S.T., Leibson C.L., Rabe K.G., Ransom J., de Andrade M., Petersen G.M. (2005). Probability of pancreatic cancer following diabetes: A populationbased study. Gastroenterology.

[5] Jin J. (2019). Screening for pancreatic cancer. J. Am. Med. Assoc..

[6] Sah R.P., Nagpal S.J., Mukhopadhyay D., Chari S.T. (2013). New insights into pancreatic cancer-induced paraneoplastic diabetes. Nat. Rev. Gastroenterol. Hepatol..

[7] Poruk K.E., Firpo M.A., Adler D.G., Mulvihill S.J. (2013). Screening for pancreatic cancer: Why, how, and who?. Ann. Surg..

[8] Chen W., Butler R.K., Lustigova E., Chari S.T., Wu B.U. (2021). Validation of the enriching new-onset diabetes for pancreatic cancer model in a diverse and integrated healthcare setting. Dig. Dis. Sci..

[9] Khan S., Safarudin R.F., Kupec J.T. (2021). Validation of the ENDPAC model: Identifying new-onset diabetics at risk of pancreatic cancer. Pancreatology.

[10] Liberati A., Altman D.G., Tetzlaff J., Mulrow C., Gøtzsche P.C., Ioannidis J.P., Clarke M., Devereaux P.J., Kleijnen J., Moher D. (2009). The PRISMA statement for reporting systematic reviews and meta-analyses of studies that evaluate healthcare interventions: Explanation and elaboration. BMJ.

[11] Whiting P.F., Rutjes A.W., Westwood M.E., Mallett S., Deeks J.J., Reitsma J.B., Leeflang M.M., Sterne J.A., Bossuyt P.M., QUADAS-2 Group (2011). QUADAS-2: A revised tool for the quality assessment of diagnostic accuracy studies. Ann. Intern. Med..

[12] Boursi B., Patalon T., Webb M., Margalit O., Beller T., Yang Y.X., Chodick G. (2022). Validation of the Enriching New-Onset Diabetes for Pancreatic Cancer Model: A Retrospective Cohort Study Using Real-World Data. Pancreas.

[13] Boursi B., Finkelman B., Giantonio B.J., Haynes K., Rustgi A.K., Rhim A.D., Mamtani R., Yang Y.-X. (2017). A clinical prediction model to assess risk for pancreatic cancer among patients with new-onset diabetes. Gastroenterology.

[14] Roy A., Sahoo J., Kamalanathan S., Naik D., Mohan P., Kalayarasan R. (2021). Diabetes and pancreatic cancer: Exploring the two-way traffic. World J. Gastroenterol..

[15] Illés D., Ivány E., Holzinger G., Kosár K., Adam M.G., Kamlage B., Zsóri G., Tajti M., Svébis M.M., Horváth V. (2020). New Onset of DiabetEs in aSsociation with pancreatic ductal adenocarcinoma (NODES Trial): Protocol of a prospective, multicentre observational trial. BMJ Open.

[16] Chari S.T., Maitra A., Matrisian L.M., Shrader E.E., Wu B.U., Kambadakone A., Zhao Y.-Q., Kenner B., Rinaudo J.A.S., Srivastava S. (2022). Early detection initiative: A randomized controlled trial of algorithm-based screening in patients with new onset hyperglycemia and diabetes for early detection of pancreatic ductal adenocarcinoma. Contemp. Clin. Trials.

[17] Schwartz N.R., Matrisian L.M., Shrader E.E., Feng Z., Chari S., Roth J.A. (2021). Potential cost-effectiveness of risk-based pancreatic cancer screening in patients with new-onset diabetes. J. Natl. Compr. Cancer Netw..

